# Vitamin D status in non-supplemented postmenopausal Taiwanese women with osteoporosis and fragility fracture

**DOI:** 10.1186/1471-2474-15-257

**Published:** 2014-07-28

**Authors:** Jawl-Shan Hwang, Keh-Sung Tsai, Yuh-Min Cheng, Wen-Jer Chen, Shih-Te Tu, Ko-Hsiu Lu, Sheng-Mou Hou, Shu-Hua Yang, Henrich Cheng, Hung Jen Lai, Sharon Lei, Jung-Fu Chen

**Affiliations:** 1 Division of Endocrinology and Metabolism, Department of Internal Medicine, Chang Gung Memorial Hospital, Chang Gung University, Linkou, Taiwan; 2 Department of Laboratory Medicine, National Taiwan University Hospital, College of Medicine, National Taiwan University, Taipei, Taiwan; 3 Department of Orthopaedic Surgery, Kaohsiung Medical University Chung-Ho Memorial Hospital, Kaohsiung, Taiwan; 4 Department of Orthopedics, Chang Gung Memorial Hospital, Chang Gung University, Linkou, Taiwan; 5 Division of Endocrinology and Metabolism, Department of Internal Medicine, Changhua Christian Hospital, Changhua, Taiwan; 6 Department of Orthopedics, Chung Shan Medical University Hospital, Taichung, Taiwan; 7 Department of Orthopedics, Shin Kong Wu Ho-Su Memorial Hospital, Taipei, Taiwan; 8 Department of Orthopaedic Surgery, National Taiwan University Hospital, Taipei, Taiwan; 9 Neurological Institute, Taipei Veterans General Hospital, Taipei, Taiwan; 10 Medical Department, Merck Sharp & Dohme (I.A.) Corp. Taiwan Branch, Taipei, Taiwan; 11 Division of Endocrinology and Metabolism, Department of Internal Medicine, Chang Gung Memorial Hospital, Chang Gung University, 123, Ta-Pei Road, Niao-Sung Hsiang, Kaohsiung, Taiwan

**Keywords:** Vitamin D, Osteoporosis, Fracture

## Abstract

**Background:**

Vitamin D is essential for calcium metabolism, Vitamin D deficiency can precipitate osteoporosis, cause muscle weakness and increase the risk of fracture. The aim of this study was to assess the prevalence of vitamin D inadequacy among non-supplemented postmenopausal women with osteoporosis and fragility fractures of the hip or vertebrae in Taiwan.

**Methods:**

This multi-center, cross-sectional, observational study analyzed the vitamin D inadequacy [defined as 25(OH) D level less than 30 ng/mL] in Taiwanese postmenopausal osteoporotic patients who suffered from a low trauma, non-pathological fragility hip or vertebral fracture that received post-fracture medical care when admitted to hospital or at an outpatient clinic.

**Results:**

A total of 199 patients were enrolled at 8 medical centers in Taiwan; 194 patients met the study criteria with 113 (58.2%) and 81 (41.8%) patients diagnosed with hip and vertebral fracture, respectively. The mean serum 25(OH) D level was 21.1 ± 9.3 ng/mL, resulting in a prevalence of vitamin D inadequacy of 86.6% of the patients.

**Conclusions:**

High prevalence of vitamin D inadequacy across all age groups was found among non-supplemented women with osteoporosis and fragility hip or vertebral fracture in Taiwan.

## Background

Osteoporosis is a progressive systemic bone disease characterized by low bone mass and microarchitecture deterioration of bone tissue, leading to increased bone fragility and susceptibility to fracture. It is recognised as a major public health problem in many countries, as well as in Taiwan [1,2]. The fractures associated with osteoporosis cause physical disability, reduced quality of life and high mortality in the aging population [3,4]. Several therapeutic options are available for the treatment or prevention of osteoporosis [5,6], and some of these drugs were proven to be efficacious and safe in Taiwanese population studies [7-10]. Among the osteoporosis management factors, vitamin D plays an important role [11,12]. Vitamin D is absorbed from food or synthesized in skin that is exposed to sunlight. The liver converts it to 25-hydroxyvitamin D [25(OH) D], which in turn is converted by the kidney to active form calcitriol 1,25 (OH)_2_D. Vitamin D increases serum calcium by promoting intestinal calcium absorption and plays a role in bone formation and resorption [13]. Synthesis of 1, 25-dihydroxyvitamin D [1, 25(OH)_2_ D] is stimulated by both PTH and hypophosphatemia. Vitamin D deficiency is associated with secondary hyperparathyroidism, which stimulates bone resorption, thus increasing the rate of bone loss and the risk of fractures [14]. In addition, vitamin D deficiency is associated with muscle weakness and postural instability, leading to an increased risk of falls [15], which may lead to fractures. However, vitamin D status associated with fractures has not been investigated in the Taiwanese population. The objective of this study was to evaluate the prevalence of vitamin D inadequacy among postmenopausal women with osteoporosis and fragility fractures of the hip or vertebrae in Taiwan.

## Methods

### Study design

This multi-center, cross-sectional, observational study was conducted in 8 medical centers in Taiwan. Among hip fracture patients, only those requiring hospitalization for post-fracture medical treatment were enrolled in this study while vertebral fracture patients were enrolled from an outpatient clinic, for a 12-month period between September 1, 2010 and September 1, 2011. The study protocol was approved by the Institutional Review Board of Chang Gung Memorial Hospital Linkou and Kaohsiung, National Taiwan University Hospital, Kaohsiung Medical University Chung-Ho Memorial Hospital, Changhua Christian Hospital, Chung Shan Medical University Hospital, Shin Kong Wu Ho-Su Memorial Hospital, and Taipei Veterans General Hospital, prior to initiation of the study and all patients gave written informed consent before any study procedure was performed. The study was conducted in compliance with the current revision of the Helsinki Declaration and in accordance with the Good Clinical Practice guidelines.

### Study subjects

The centers recruited postmenopausal women aged 50 years and over with a recent low-trauma fragility vertebral fracture at outpatient clinic or hip fracture inpatient care. Low-trauma fractures were defined as fractures resulting from falls from standing height. Recent fracture was defined as hip fracture or related clinical signs/symptoms occurring within 30 days of the study enrollment date; vertebral fracture or related clinical signs/symptoms occurring within 3 months of the study enrollment date. Fracture was confirmed by radiograph or X-ray report; categorization of vertebral fracture was assessed centrally.

Patients who had secondary osteoporosis or other diseases which could affect bone metabolism, significant hepatic or renal diseases [defined as serum alanine aminotransferase (ALT) > 3 times upper limit of normal (ULN), and creatinine (Cr) > 1.6 mg/dL], malignant neoplasm, major trauma (e.g. automobile accident) or recent use of drugs known to affect bone metabolism were excluded. Patients who participated in a clinical trial for osteoporosis within the past 3 years, had low trauma fracture of sites other than the hip or vertebrae, mentally or legally incapacitated or unable to answer health-related questions were also excluded as well as patients with premature or surgical menopause, e.g. oophorectomy due to ovarian cancer, or patients who have regularly been receiving 2800 IU vitamin D supplement per week or 400 IU vitamin D supplement per day at study enrollment.

#### Study procedures

Patients were required to have a physical examination, lateral radiographs of the thoracic and lumbar spine for vertebral fracture patients group, documentation of past medical history and concomitant medications that affect vitamin D metabolism, any use of anti-osteoporotic therapies, pre-fracture daily activity and health-related questionnaire. A single, non-fasting blood sample to assess 25 (OH) D and other biochemical tests were performed in the study visit. Vitamin D inadequacy was defined as serum 25 (OH) D level less than 30 ng/mL. Considering prior vitamin D studies definitions [12,16-18], we further dissected vitamin D inadequacy into insufficiency as 25(OH) D 10 to 30 ng/mL and deficiency as 25(OH) D < 10 ng/mL. In addition to this, a cut-point of 25 (OH) D less than 20 ng/mL was also analyzed to allow comparison with some recent studies.

The radiographs were performed according to a standardized protocol. A visual semiquantitative grading of vertebral fractures was performed, based on the criteria of Genant et al., by a radiologist in a centralized way. Bone mineral density (BMD) measured by dual energy X-ray absorptiometry of lumbar spine and/or hip were recorded.

Biochemical measurements were standardized using central laboratory tests method.

Serum calcium, phosphate, and creatinine were measured by automated standard laboratory methods. Intact PTH (i-PTH) was measured by ADVIA Centaur chemilluminescence instruments (SIEMENS Healthcare, Tarrytown, New York), 25-OH Vitamin D Total by chemilluminescence (Diasorin, MN, USA). The inter-assay variations for the three-level controls for assays i-PTH were 6.55%, 6.02% and 5.0%, and 25 (OH) D two level controls 6.06% and 6.15%, respectively. Bone turnover markers were measured in all patients with Bone Alkaline Phosphatase (BAP), by chemilluminescence using Beckman Access II (Fullerton, California) with inter-assay variations for the markers were 4.62% and 5.13%.

### Statistical analysis

Data management and analysis were performed using the SAS 8.2 (SAS Institute Inc., Cary, NC, USA) and SPSS 20.0 (IBM., USA). The prevalence of vitamin D inadequacy was analyzed; insufficiency and deficiency were also summarized. Age and fracture site stratified prevalence of vitamin D insufficiency and deficiency were also calculated. Data were presented as mean ± SD for continuous variables, the number and the proportions were shown for categorical variables.

Student t test, Wilcoxon rank sum test or Analysis of variance (ANOVA) were used to examine the differences between/among groups for continuous variables upon the data distribution, and chi-square test or Fisher’s exact test were used for categorical variables. Meanwhile, Pearson correlation was used to assess the correlation between serum vitamin D levels versus fracture risk factors in these patients. All significance tests were two-tailed with *p* = 0.05.

## Results

**Figure 1:**
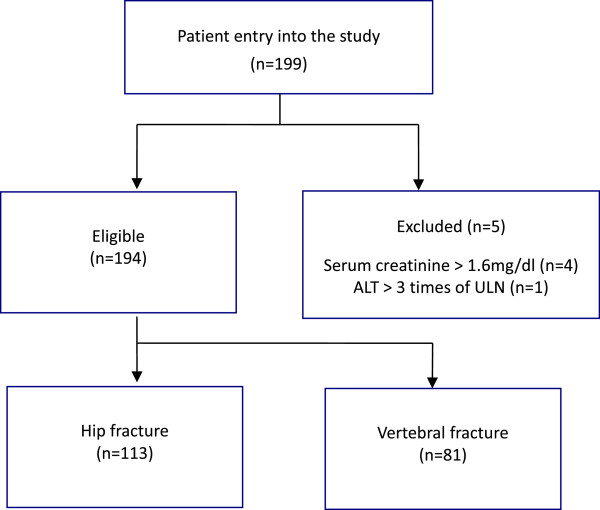
Patient distribution in the study.

A total of 199 subjects were evaluated for inclusion with 194 subjects meeting all of the eligibility criteria as shown in Figure 1. Five subjects were excluded due to renal or liver impairment (4 subjects with Cr > 1.6 mg/dL and 1 subject with ALT > 3 times ULN). The demographics and characteristics of the patients are shown in Table 1. Overall, the average age was 77.6years (range: 52 to 103 years) and the mean BMI was 23.9 kg/m^2^. The mean years since menopause were 27.4 with a minimum of 5 years and maximum of 51 years. There was no difference of BMD at any site between the hip and vertebral fractures group.

**Table 1:** Patients’ characteristics

	**Hip fracture (n = 113)**	**Vertebral fracture (n = 81)**	***p*-value**
Age (years), mean ± SD	79.5 ± 9.3	74.6 ± 8.5	<0.01
Height (cm), mean ± SD	152.9 ± 5.9	150.7 ± 6.1	0.015
Weight (kg), mean ± SD	55.0 ± 10.1	55.6 ± 10.8	0.688
BMI (kg/m2), mean ± SD	23.5 ± 4.1	24.4 ± 3.9	0.153
Year of menopause, mean ± SD	28.3 ± 10.5	26.1 ± 8.5	0.156
BMD (T-score), mean ± SD			
Spine L1-4	-2.7 ± 1.6	-3.0 ± 1.4	0.383
Femoral neck	-3.2 ± 0.9	-3.2 ± 1.0	0.986
Total hip	-2.9 ± 1.0	-2.6 ± 1.1	0.149
Co-morbidity (n,%)			
Hypertension	72 (63.7%)	42 (51.9%)	0.033
Diabetes	27 (23.9%)	17 (21.0%)	0.634
Stroke	5 (4.4%)	5 (6.2%)	0.742
Parkinson’s disease	2 (1.8%)	1 (1.2%)	1.000
Calcium and Vitamin D containing food (n,%)			
Milk	73 (64.6%)	44 (54.3%)	0.149
Yolk	95 (84.1%)	66 (81.5%)	0.773
Cod-liver oil/fatty fish/mushrooms	53 (46.9%)	22 (27.2%)	0.005

The patient characteristics showed little difference between the hip and vertebral fracture group. Among 113 hip and 81 vertebral fracture women, the average age was 79.5years in hip fracture women while it was 74.6 years in vertebral fracture women. Regarding the co-morbidity survey, only hypertension revealed a statistical difference (*p* = 0.033) between the groups, with 63.7% of the hip fracture women diagnosed with hypertension compared to 51.9% of the vertebral fracture group. Also, food composition in terms of daily vitamin D source showed some differences between groups. Among several types of calcium-rich and vitamin-containing food, including milk, yolk, cod-liver oil, fatty fish, and mushroom, the hip fracture group had higher proportion of women taking cod-liver oil and eating more fatty fish and mushrooms (46.9% vs. 27.2%) than the vertebral fracture group.

Overall, the mean level of 25(OH) D was 21.1 ± 9.3 ng/mL, ranging from 4.0 to 57.5 ng/mL (median: 20.0, Q1:15.7, Q3:26.2). The prevalence of vitamin D inadequacy with serum 25(OH) D level lower than 30 ng/mL was 86.6% of the subjects. Further dissection, vitamin D insufficiency [25(OH) D 10–30 ng/mL] prevalence was 77.8% while vitamin D deficiency [25(OH) D < 10 ng/mL] was 8.8% of the subjects (Table 2). Additionally, when using another cut-off point of 25(OH) D < 20 ng/mL, the prevalence was 49.5%, as shown in Table 3.

**Table 2:** Distribution of serum 25 (OH) D level in 194 enrolled subjects

	**n (%)**	**Mean (SD)**	**Range (Min, Max)**
Adequacy [≥30 ng/ml]	26 (13.4%)	37.6 (7.2)	(30.0, 57.5)
Inadequacy [<30 ng/ml]	168 (86.6%)	18.6 (6.5)	(4.0, 29.7)
Insufficiency [10–30 ng/ml]	151 (77.8%)	20.0 (5.3)	(10.0, 29.7)
Deficiency [<10 ng/ml]	17 (8.8%)	6.4 (1.6)	(4.0, 9.3)

**Table 3:** Prevalence of serum 25(OH) D levels using three cut-off points

	**<10 ng/ml**	**<20 ng/ml**	**<30 ng/ml**
Overall (n = 194)	17 (8.8%)	96 (49.5%)	168 (86.6%)
Hip fracture (n = 113)	12 (10.6%)	60 (53.1%)	99 (87.6%)
Vertebral fracture (n = 81)	5 (6.2%)	36 (44.4%)	69 (85.2%)

NS between fracture types.

There was no statistically significant difference in the mean 25(OH) D levels between hip and vertebral fractures subjects, 20.5 vs. 22.0 ng/mL, respectively (*p* = 0.279; Table 4). In terms of serum markers, in the hip fracture group, there were significantly higher mean i-PTH levels (45.7 vs. 35.8 pg/mL), lower mean serum calcium (8.2 vs. 8.9 mg/dL), lower mean serum phosphorus (3.5 vs. 4.2 mg/dL) and lower mean BAP levels (13.0 vs. 18.2 mg/dL) compared to vertebral fracture group (Table 4). Serum creatinine and alanine aminotransferase were similar between the groups. The mean vitamin D levels were slightly higher but no statistical difference in summer and fall than in winter and spring season.

**Table 4:** Biochemical parameters

	**Hip fracture (n = 113)**	**Vertebral fracture (n = 81)**	**p-value**
Serum 25(OH) D (ng/ml)	20.5 ± 9.9	22.0 ± 8.4	0.279
i-PTH(pg/ml)	45.7 ± 29.2	35.8 ± 19.4	0.005
Calcium (mg/dl)	8.2 ± 0.6	8.9 ± 0.5	0.000
Phosphorus (mg/dl)	3.5 ± 1.0	4.2 ± 2.2	0.004
BAP (mg/dl)	13.0 ± 6.9	18.2 ± 9.3	0.000

Data are expressed as mean ± standard deviation.

When analyzing the mean serum 25(OH) D levels and prevalence using three cut-off points (<30, <20 and < 10 ng/mL) by different age groups (<60; 60 ~ 69; 70 ~ 79; 80 ~ 84; ≥85 years), the results were consistent across all age groups with no statistical difference in the vitamin D levels or proportion of patients with vitamin D levels lower than 30 ng/mL, 20 ng/mL or 10 ng/mL in any of the age groups (Table 5).

**Table 5:** Mean serum 25(OH) D levels and prevalence in different age groups

	**<10 ng/ml**	**<20 ng/ml**	**<30 ng/ml**	**25(OH) D level (mean ± SD)**
<60 y/o (n = 2)	0 (0%)	0 (0%)	2 (100%)	23.3 ± 1.7
60 ~ 69 y/o (n = 36)	3 (8.3%)	17 (47.2%)	29 (80.6%)	22.5 ± 10.4
70 ~ 79 y/o (n = 64)	4 (6.3%)	33 (51.6%)	61 (95.3%)	20.4 ± 7.9
80 ~ 84 y/o (n = 41)	3 (7.3%)	24 (58.5%)	34 (82.9%)	21.5 ± 10.3
≥85 y/o (n = 46)	7 (15.2%)	21 (45.7%)	38 (82.6%)	20.2 ± 9.3

NS among age groups.

## Discussion

This is the first study to examine the prevalence of vitamin D inadequacy among osteoporotic women with fragility hip or vertebral fracture in Taiwanese population. The results of this study have shown a high prevalence of vitamin D inadequacy (insufficiency and deficiency) across all age groups among women with fragility hip or vertebral fracture in Taiwan. Some studies conducted in Taiwan and Asia have shown nearly half of healthy, middle-aged and elderly women had vitamin D inadequacy, nearly one-third of adults with frailty syndrome had vitamin D insufficiency [19,20] and inadequacy levels are more common in some Asian countries than in other regions [21].

International Osteoporosis Foundation recommends that a minimum level of 30 ng/mL 25(OH) D is necessary in older adults to reduce the risk of falls and fractures [15]. Some guidelines suggest a 25(OH) D level of at least 20 ng/mL to promote intestinal calcium absorption, avoid secondary hyperparathyroidism and minimize the risk of fracture [22]. In our study, the prevalence of vitamin D insufficiency, 25(OH) D level lower than 30 ng/mL, was 86.6% and the prevalence of 25(OH) D lower than 20 ng/mL was 49.5%. As compared to the National Health and Nutrition Examination Survey (NHANES) 2005 to 2006, where 41.6% of adults had 25(OH) D below 20 ng/mL [23], in the current study we have found a higher proportion of vitamin D insufficiency (49.5%, vs. 41.6%), probably due to inclusion of subjects with confirmed fragility fracture either admitted to hospital or from outpatient clinics and due to exclusion of patients taking vitamin D supplementation regularly (2800 IU vitamin D per week or 400 IU vitamin D per day). The results were consistent with the previous findings that low vitamin D levels were associated with a high hip fracture rate [24] and other osteoporotic fractures, including vertebral and wrist fractures [25]. One recent study has shown that among patients admitted to a rehabilitation facility, 49.1% had 25(OH) D levels below 20 ng/mL and 83% lower than 30 ng/mL [26], which is consistent with the current study results. Another study in the literature has shown 68% of hypo-vitaminosis D in an orthogeriatric rehabilitation ward within 7 days after hip fracture [27].

Vitamin D plays an important role in protein synthesis and the regulation of calcium transport which affects muscle strength as well as the balance among neurons and muscle. This might be the reason why vitamin D deficiency has been associated with muscle weakness and postural instability, leading to an increased risk of falls, which may lead to fractures [28,29]. Low vitamin D levels are also associated with decline in physical performance in the elderly [30].

The relationship of 25(OH) D level and i-PTH is consistent with previous reports that the synthesis and metabolism of vitamin D is coupled to calcium homeostasis and is regulated by PTH, serum calcium and phosphorus levels [12]. When hypocalcaemia occurs, serum PTH concentration increases and enhances reabsorption of calcium. In our study, serum 25(OH) D was inversely correlated with serum i-PTH (*r* = -0.22, *p* < 0.01).

The possible explanations for the high prevalence of vitamin D insufficiency in this cohort of Taiwanese women could be several. First, the Recommended Dietary Allowance (RDA) of vitamin D for the elderly is 800 international units per day after age 71 [22], but only 17.5% subjects have vitamin D supplements in daily life and larger-dose preparations of ergocalciferol or cholecalciferol are rare in Taiwan. Second, the consumption of fatty fish, vitamin D-fortified foods and vitamin D supplements also influence the status of vitamin D insufficiency [31,32]. This is the largest source of dietary vitamin D in many countries, but fortified milk, cereals and bread products with vitamin D in Taiwan are rarely found. Therefore, vitamin D from dietary source might be limited due to less options and lower availability of vitamin D-containing food in addition to decreased intestinal absorption of vitamin D in elderly patients. Third, the vitamin D status depends on the available amount of ultraviolet light from sunlight which varies with latitude and season; use of sunscreen and clothing; and amount of sunlight exposure which increases vitamin D concentration [33]. Although Taiwan is situated at latitude between 23 and 25 degrees, sun-avoidance behavior, skin covering and the use of sunscreen products in the Taiwanese population interfere with production of vitamin D in the skin. In addition, the serum vitamin D level has been shown to decrease with aging, with the skin of those older than 70 years of age being less efficient in converting 7- dehydrocholesterol into vitamin D as compared to younger individuals, impairing dermal biosynthesis of vitamin D [34].

There are some limitations of the current study. The sample size of this study (n = 194) was relatively small although well spread and distributed throughout the island. Also, this study did not include healthy subjects without fractures or male gender as normal controls. Another limitation was the study design which did not allow us to collect vitamin D levels at the exact time of fracture; however, it was not expected that levels of vitamin D would vary significantly in the short period of time stipulated for blood collection after fracture.

## Conclusions

High prevalence of vitamin D insufficiency and deficiency was found among post-menopausal women with osteoporosis and fragility hip or vertebral fracture in the Taiwanese population. Recommendations and public education for postmenopausal women with osteoporosis to receive adequate vitamin D supplementation should be reinforced.

## Competing interests

Dr. Hung-Jen Lai & Ms. Sharon Lei are the employees of Merck Sharp & Dohme (I.A.) Corp. Taiwan Branch. All other authors declare that they have no competing interests.

## Authors’ contributions

JSH, KST, HJL, SL and JFC were responsible for study conception and design, drafting the manuscript. YMC, WJC, STT, KHL, SMH, SHY and HC were responsible for data analysis. All authors were responsible for discussing and interpreting the results and writing the final version to be published. All authors read and approve the final manuscript.

## Pre-publication history

The pre-publication history for this paper can be accessed here:

http://www.biomedcentral.com/1471-2474/15/257/prepub

## References

[B1] Cummings SR Melton LJ Epidemiology and outcomes of osteoporotic fractures Lancet 2002 359 1761 1767 12049882 10.1016/S0140-6736(02)08657-9

[B2] Tsai KS Tai TY Epidemiology of osteoporosis in Taiwan Osteoporos Int 1997 7 Suppl 3 96 98 10.1007/BF03194352 9536312

[B3] Center JR Nguyen TV Schneider D Sambrook PN Eisman JA Mortality after all major types of osteoporotic fracture in men and women: an observational study Lancet 1999 353 878 882 10093980 10.1016/S0140-6736(98)09075-8

[B4] Cooper C The crippling consequences of fractures and their impact on quality of life Am J Med 1997 103 2A 12S 17S 9302893 10.1016/s0002-9343(97)90022-x

[B5] Cranney A Guyatt G Griffith L Wells G Tugwell P Rosen C Osteoporosis Methodology Group and The Osteoporosis Research Advisory Group Meta-analyses of therapies for postmenopausal osteoporosis. IX: Summary of meta-analyses of therapies for postmenopausal osteoporosis Endocr Rev 2002 23 570 578 12202472 10.1210/er.2001-9002

[B6] Chapuy MC Arlot ME Duboeuf F Brun J Crouzet B Arnaud S Delmas PD Meunier PJ Vitamin D3 and calcium to prevent hip fractures in the elderly women N Engl J Med 1992 327 23 1637 1642 1331788 10.1056/NEJM199212033272305

[B7] Hwang JS Tu ST Yang TS Chen JF Wang CJ Tsai KS Teriparatide vs. calcitonin in the treatment of Asian postmenopausal women with established osteoporosis Osteoporos Int 2006 17 373 378 16421647 10.1007/s00198-005-2002-5

[B8] Hwang JS Chen JF Yang TS Wu DJ Tsai KS Ho C Wu CH Su SL Wang CJ Tu ST The effects of strontium ranelate in Asian women with postmenopausal osteoporosis. [Erratum appears in Calcif Tissue Int. 2009 Apr;84(4):334] Calcif Tissue Int 2008 83 308 314 18843436 10.1007/s00223-008-9180-z

[B9] Hwang JS Liou MJ Ho C Lin JD Huang YY Wang CJ Tsai KS Chen JF The effects of weekly alendronate therapy in Taiwanese males with osteoporosis J Bone Miner Metab 2010 28 328 333 20012918 10.1007/s00774-009-0136-9

[B10] Hwang JS Chin LS Chen JF Yang TS Chen PQ Tsai KS Leung PC The effects of intravenous zoledronic acid in Chinese women with postmenopausal osteoporosis J Bone Miner Metab 2011 29 328 333 20922438 10.1007/s00774-010-0223-y

[B11] Lips P van Schoor NM The effect of vitamin D on bone and osteoporosis Best Pract Res Clin Endocrinol Metab 2011 25 585 591 21872800 10.1016/j.beem.2011.05.002

[B12] Dawson-Hughes B Mithal A Bonjour JP Boonen S Burckhardt P Fuleihan GE Josse RG Lips P Morales-Torres J Yoshimura N IOF position statement: vitamin D recommendations for older adults Osteoporos Int 2010 21 1151 1154 20422154 10.1007/s00198-010-1285-3

[B13] Lips P Vitamin D, physiology Prog Biophys Mol Biol 2006 92 4 8 16563471 10.1016/j.pbiomolbio.2006.02.016

[B14] Lips P Bouillon R van Schoor NM Vanderschueren D Verschueren S Kuchuk N Milisen K Boonen S Reducing fracture risk with calcium and vitamin D Clin Endocrinol (Oxf) 2010 73 277 285 20796001 10.1111/j.1365-2265.2009.03701.x

[B15] Pfeifer M Begerow B Minne HW Vitamin D and muscle function Osteoporos Int 2002 13 187 194 11991436 10.1007/s001980200012

[B16] Rosen CJ Clinical practice. Vitamin D insufficiency N Engl J Med 2011 364 248 254 21247315 10.1056/NEJMcp1009570

[B17] Mithal A Wahl DA Bonjour JP Burckhardt P Dawson-Hughes B Eisman JA El-Hajj FG Josse RG Lips P Morales-Torres J IOF Committee of Scientific Advisors (CSA) Nutrition Working Group Global vitamin D status and determinants of hypovitaminosis D. [Erratum appears in Osteoporos Int. 2009 Nov; 20 (11):1821] Osteoporos Int 2009 20 1807 1820 19543765 10.1007/s00198-009-0954-6

[B18] El-Hajj FG Vitamin D deficiency in the Middle East and its health consequences Clin Rev Bone Miner Metab 2009 7 77 93 10.1007/s12018-009-9038-6 PMC4126271 25110467

[B19] Tsai KS Hsu SH Cheng JP Yang RS Vitamin D stores of urban women in Taipei: effect on bone density and bone turnover, and seasonal variation Bone 1997 20 371 374 9108358 10.1016/s8756-3282(97)00010-0

[B20] Chang CI Chan DC Kuo KN Hsiung CA Chen CY Vitamin D insufficiency and frailty syndrome in older adults living in a Northern Taiwan community Arch Gerontol Geriatr 2010 50 Suppl 1 17 21 10.1016/S0167-4943(10)70006-6 20171450

[B21] Lim SK Kung AW Sompongse S Soontrapa S Tsai KS Vitamin D inadequacy in postmenopausal women in Eastern Asia Curr Med Res Opin 2008 24 99 106 18028585 10.1185/030079908x253429

[B22] Holick MF Binkley NC Bischoff-Ferrari HA Gordon CM Hanley DA Heaney RP Murad MH Weaver CM Society E Evaluation, treatment, and prevention of vitamin D deficiency: an Endocrine Society clinical practice guideline J Clin Endocrinol Metab 2011 96 1911 1930 21646368 10.1210/jc.2011-0385

[B23] Forrest KY Stuhldreher WL Prevalence and correlates of vitamin D deficiency in US adults Nutr Res 2011 31 48 54 21310306 10.1016/j.nutres.2010.12.001

[B24] Cauley JA Lacroix AZ Wu L Horwitz M Danielson ME Bauer DC Lee JS Jackson RD Robbins JA Wu C Stanczyk FZ LeBoff MS Wactawski-Wende J Sarto G Ockene J Cummings SR Serum 25-hydroxyvitamin D concentrations and risk for hip fractures Ann Intern Med 2008 149 242 250 18711154 10.7326/0003-4819-149-4-200808190-00005 PMC2743412

[B25] Van Schoor NM Visser M Pluijm SMF Kuchuk N Smit JH Lips P Vitamin D deficiency as a risk factor for osteoporotic fractures Bone 2008 42 260 266 18289505 10.1016/j.bone.2007.11.002

[B26] Shinchuk LM Morse L Huancahuari N Arum S Chen TC Holick MF Vitamin D deficiency and osteoporosis in rehabilitation inpatients Arch Phys Med Rehabil 2006 87 904 908 16813775 10.1016/j.apmr.2006.03.009

[B27] Sahota O Gaynor K Harwood RH Hosking DJ Hypovitaminosis D and 'functional hypoparathyroidism'-the NoNoF (Nottingham Neck of Femur) study Age Ageing 2001 30 467 472 11742774 10.1093/ageing/30.6.467

[B28] Bischoff-Ferrari HA Dietrich T Orav EJ Hu FB Zhang Y Karlson EW Dawson-Hughes B Higher 25-hydroxyvitamin D concentrations are associated with better lower-extremity function in both active and inactive persons aged > or =60 y Am J Clin Nutr 2004 80 752 758 15321818 10.1093/ajcn/80.3.752

[B29] Visser M Deeg DJ Lips P Longitudinal Aging SA Low vitamin D and high parathyroid hormone levels as determinants of loss of muscle strength and muscle mass (sarcopenia): the Longitudinal Aging Study Amsterdam J Clin Endocrinol Metab 2003 88 5766 5772 14671166 10.1210/jc.2003-030604

[B30] Wicherts IS van Schoor NM Boeke AJ Visser M Deeg DJ Smit J Knol DL Lips P Vitamin D status predicts physical performance and its decline in older persons J Clin Endocrinol Metab 2007 92 2058 2065 17341569 10.1210/jc.2006-1525

[B31] Holden JM Lemar LE Assessing vitamin D contents in foods and supplements: challenges and needs Am J Clin Nutr 2008 88 551S 553S 18689400 10.1093/ajcn/88.2.551S

[B32] Ross AC The 2011 report on dietary reference intakes for calcium and vitamin D Public Health Nutr 2011 14 938 939 21492489 10.1017/S1368980011000565

[B33] Binkley N Novotny R Krueger D Kawahara T Daida YG Lensmeyer G Hollis BW Drezner MK Low vitamin D status despite abundant sun exposure J Clin Endocrinol Metab 2007 92 2130 2135 17426097 10.1210/jc.2006-2250

[B34] MacLaughlin J Holick MF Aging decreases the capacity of human skin to produce vitamin D3 J Clin Invest 1985 76 1536 1538 2997282 10.1172/JCI112134 PMC424123

